# Editorial: Interview with the translational apparatus: stories of intriguing circuits and mechanisms to regulate translation in bacteria, volume II

**DOI:** 10.3389/fmicb.2023.1195257

**Published:** 2023-06-13

**Authors:** Anna Maria Giuliodori, Paola Londei, Stefano Marzi

**Affiliations:** ^1^School of Biosciences and Veterinary Medicine, University of Camerino, Camerino, Italy; ^2^Department of Molecular Medicine, University of Rome Sapienza, Rome, Italy; ^3^Architecture et Reactivite de l'ARN, CNRS 9002, Universite de Strasbourg, Strasbourg, France

**Keywords:** translation, regulation, gene expression, cold adaptation, nutrient balance

Bacteria are continuously subjected to a variety of stressors, including fluctuations in temperature, pH, nutrient availability, salt and oxygen concentration, which can cause genetic reprogramming and alteration in cell metabolism, growth and survival. By modulating protein levels, translational control provides a swift and accurate response to cope with and react rapidly to these diverse stressors.

In the previous Research Topic of “*Interview with the Translational Apparatus: Stories of Intriguing Circuits and Mechanisms to Regulate Translation in Bacteria*” we compiled several manuscripts that highlighted the molecular diversity of bacterial translation regulation and the various techniques employed to study it (Giuliodori and Marzi, 2021). For Volume II, we have included four additional original research articles that examine the regulation of translation in *Escherichia coli* under stressful conditions such as cold acclimatization, oxidative stress, and variations of intracellular phosphate concentration. These articles identify and describe the different elements that play a role in regulating gene expression at the translational level in one of the most extensively studied model organisms. Understanding the mechanisms by which bacteria respond to stress can help to better understand their biology, as well as to develop new strategies for controlling bacterial populations in various environments.

Enterobacteriaceae frequently encounter temperature changes as they move from the intestinal to external habitats. The sudden decrease in temperature triggers the cold-shock response, which involves an adaptation period, during which mesophilic bacteria, such as *E. coli*, stop growing and block or reduce the synthesis of many macromolecules. This phase sees a temporarily increase in the expression of a specific group of genes, referred to as cold-shock genes. Unlike other regulons, cold-shock gene expression is mainly regulated post-transcriptionally, either by modulating transcript half-life or by controlling translation efficiency (for a review see Giuliodori, 2016; Zhang and Gross, 2021).

Giuliodori et al. investigate the role of CspA, a key cold-shock protein in *E. coli*, during translation at low temperature, using molecular biology techniques such as reconstituted translation systems and probing methods. In a previous study, the authors demonstrated that the translation of *cspA* mRNA is facilitated by a thermosensor element that opens the ribosome binding site during cold-shock and makes it less accessible at 37°C (Giuliodori et al., 2010). The present research builds upon these findings and demonstrates that CspA specifically promotes the translation of *cspA* mRNA folded in the conformation that is less accessible to the ribosome. The mechanism by which CspA promotes translation is by facilitating the ribosomes' progression from translation initiation to elongation (Giuliodori et al.). The study suggests that this structure-dependent mechanism may also occur for other cold-shock genes targeted by CspA.

Bacteria inhabiting the intestine often also experience changes in the intracellular pool of phosphate (Pi) due to dietary variations and the aforementioned transition from the intra to the extra-intestinal environment. To respond rapidly to these fluctuations, bacteria have developed a regulatory circuit consisting of a phosphate-specific transport system, encoded by the *pstSCAB-phoU* genes, and the PhoR-PhoB two-component system. The PhoR transmembrane sensor histidine kinase detects Pi limitation and activates PhoB, the response regulator, which controls the expression of the genes containing a Pho box (for a review see Gardner and McCleary, 2019). Furthermore, Pi starvation activates the synthesis of (p)ppGpp, resulting in a decrease in rRNA and tRNA synthesis.

The article from Espinosa et al., investigates factors and mechanisms that downregulate protein synthesis in response to phosphate starvation in *E. coli*. Through the use of radiolabeling, the authors show that phosphate starvation leads to a decrease in total cellular RNA, primarily due to a reduction in rRNA, while protein synthesis continues, albeit at a reduced rate, before eventually stopping. To understand why this occurs, the authors compared the protein synthesis rate in *E. coli* cells before and after phosphate starvation, with and without the overproduction of an induced and long-lived mRNA that is highly translated. The data analysis suggests that the reduced amounts of mRNAs is the limiting factor for protein synthesis during phosphate starvation, which may be an adaptive response to conserve resources.

Bacteria have developed defense systems against reactive oxygen species (ROS) such as superoxide (O_2_-) and hydrogen peroxide (H_2_O_2_) to protect their cellular components. Oxidative stress activates various stress responses, which depend on the type of ROS and microorganism (for a review see Fasnacht and Polacek, 2021). In gram-negative bacteria, H_2_O_2_ typically induces the expression of the transcriptional factor OxyR, which regulates about thirty stress genes. In *E. coli*, an increase in O_2_- activates SoxR and then SoxS, which controls the transcription of about 25 genes. Alongside these regulons, oxidative stress also affects tRNA, ribosomes, and factors, resulting in a significant impact on the traditional translation process.

Leiva et al. reported that oxidative stress affects both translation initiation and elongation in *E. coli*. To examine the impact of oxidative stress on translation, the authors developed 61 GFP reporter constructs, each containing a unique sequence of four repeated amino acids inserted between the fourth and fifth codons of the GFP coding region. The system enabled the authors to demonstrate that codon choice influences GFP translation when *E. coli* K-12 cells are grown in minimal media, but not when they are exposed to oxidative stress. In this latter condition GFP expression is overall reduced and the rate of translation initiation influences the speed of protein synthesis more than under normal conditions.

The original research carried out by Chen et al. aims to explore the impact of different 5′UTR sequences on gene expression regulation in *E. coli*. It is known that mRNA 5′ UTR are regulatory-hubs switching on and off translation or modulating the expression of several genes. The team assessed both rate of synthesis and mRNA stability of a large collection of constructs containing synthetic 5′UTR sequences, differing at the level of the ribosome binding site and linked to various reporter genes. Their findings showed that the predicted theoretical translation initiation rate did not accurately reflect protein levels for most of the synthetic 5′UTRs. Furthermore, several 5′UTRs were found to affect mRNA stability in a reporter gene-dependent manner.

## Author contributions

All authors listed have made a substantial, direct, and intellectual contribution to the work and approved it for publication.
